# Mother–child dyads of overnutrition and undernutrition in sub-Saharan Africa

**DOI:** 10.1186/s41043-023-00479-y

**Published:** 2024-01-02

**Authors:** Joshua Okyere, Irene Esi Donkoh, Abdul-Aziz Seidu, Bright Opoku Ahinkorah, Richard Gyan Aboagye, Sanni Yaya

**Affiliations:** 1 Department of Population and Health, University of Cape Coast, Cape Coast, Ghana; 2 Department of Nursing, College of Health Sciences, Kwame Nkrumah University of Science and Technology, Kumasi, Ghana; 3 Department of Medical Laboratory Science, University of Cape Coast, Cape Coast, Ghana; 4 Centre for Gender and Advocacy, Takoradi Technical University, Takoradi, Ghana; 5 College of Public Health, Medical and Veterinary Sciences, James Cook University, Townsville, Australia; 6 School of Clinical Medicine, University of New South Wales Sydney, Sydney, Australia; 7 School of Public Health, Faculty of Health, University of Technology Sydney, Sydney, Australia; 8 Department of Family and Community Health, Fred N. Binka School of Public Health, University of Health and Allied Sciences, Hohoe, Ghana; 9 School of International Development and Global Studies, University of Ottawa, Ottawa, Canada; 10 The George Institute for Global Health, Imperial College London, London, UK

**Keywords:** Stunting, Underweight, Wasting, Undernutrition, Sub-Saharan Africa, Demographic and Health Survey

## Abstract

**Background:**

Malnutrition remains one of the major public health concerns globally. To achieve the Sustainable Development Goal 2 which seeks to ensure that hunger and malnutrition are reduced by 2030, it is imperative to ascertain the factors influencing their occurrence. This study examined the prevalence and factors associated with mother–child dyads of overnutrition and undernutrition in sub-Saharan Africa.

**Methods:**

Demographic and Health Survey data from 25 sub-Saharan African countries were used for the study. The sample was made up of 125,280 mother–child dyads. Descriptive analysis was performed to determine the prevalence of overweight or obese mother (OWOBM) with a stunted child (OWOBM-SC), OWOBM with an underweight child (OWOBM-UC), OWOBM with a wasted child (OWOBM-WC), and OWOBM with any form of child’s undernutrition indicators (OWOBM-SUWC). Multilevel regression models were developed to examine the factors associated with these indicators. The results were presented using an adjusted odds ratio (AOR) with their respective 95% confidence interval (CI).

**Results:**

Higher likelihood of OWOBM-SUWC was found among women aged 45–49 [AOR 2.20, 95% CI 1.70, 2.85], those with primary [AOR 1.32, 95% CI 1.21, 1.44] or secondary education [AOR 1.21, 95% CI 1.09, 1.35], and divorced women [AOR 1.32, 95% CI 1.02, 1.73]. However, lower odds of OWOBM-SUWC were observed among women who were working [AOR 0.82, 95% CI 0.76, 0.89] and those breastfeeding [AOR 0.75, 95% CI 0.70, 0.82]. The odds of OWOBM-SUWC was lower among females compared to male children [AOR 0.85, 95% CI 0.80, 0.90]. Compared to children aged <1 year, children of all other age groups were more likely to have OWOBM-SUWC. Other child characteristics significantly associated with OWOBM-SUWC were low birth weight [AOR 1.50, 95% CI 1.32, 1.71], having diarrhea [AOR 1.13, 95% CI 1.04, 1.24], and higher birth order [AOR 1.37, 95% CI 1.13, 1.66]. Children whose mothers used unimproved toilet facilities [AOR 0.90, 95% CI 0.83, 0.98], those who lived in rural areas [AOR 0.79, 95% CI 0.71, 0.87], and children from the Central [AOR 0.55, 95% CI 0.46, 0.65], Eastern [AOR 0.44, 95% CI 0.38, 0.52] and Western [AOR 0.76, 95% CI 0.65, 0.89] sub-Saharan Africa were less likely to have OWOBM-SUWC.

**Conclusion:**

Combination of child, maternal, and contextual factors could explain mother–child dyads of overnutrition and undernutrition in sub-Saharan Africa. Addressing this situation requires multidimensional policies and interventions that empower women through education and economic engagement. The observed sub-regional differences in policies and commitments related to addressing malnutrition suggest the need for comprehensive and coordinated efforts to implement and strengthen multisectoral comprehensive nutrition plans across sub-Saharan Africa. Sharing best practices and lessons learned can help improve the effectiveness and comprehensiveness of nutrition interventions and contribute to reducing the prevalence of malnutrition.

**Supplementary Information:**

The online version contains supplementary material available at 10.1186/s41043-023-00479-y.

## Introduction

Malnutrition is a major public health problem globally. The devastating consequences of malnutrition has made it an important component of the United Nations' Sustainable Development Goals (SDG 2) [1]. With food and nutritional security as key factors contributing to malnutrition, the world races to achieve SDG 2, which seeks to end hunger, achieve food security and improved nutrition and promote sustainable agriculture by 2030 [1]. In 2021, there were 38.9 million overweight children, 149.2 and 45.4 million children under the age of five were wasted and stunted, respectively, while 462 million adults were underweight [2]. Recent evidence suggests that a large proportion of low-and middle-income countries (LMICs) face the double burden of malnutrition (DBM), a state characterized by the coexistence of undernutrition (ie, micronutrient deficiencies, underweight, and childhood stunting, and wasting) and overweight, obesity, and diet-related non-communicable diseases [3]. Also, while other LMICs are challenged with DBM [3], others are faced with the existence of overnutrition, undernutrition, and micronutrient deficiencies, a term commonly referred to as triple burden of malnutrition (TBM) [4]. Globally, the prevalence of malnutrition at the household level averages less than 10%, but it continues to increase particularly in LMICs [5].

Despite improvement in childhood nutritional status globally, LMICs still face malnutrition as a public health challenge [6]. This is extremely common in most sub-Saharan African countries where between 22% to 66% of children in every household are either underweight, wasted, stunted, or overweight [7]. A more severe form of nutritional burden is the disparity in the nutritional status of the mother–child dyad. There exists a common belief that since mothers and their children share access to food, hygiene products, and other resources, there should be less of a chance that their nutritional statuses differ [8].

The mother–child DBM is a recognized global concern because while uncontrolled obesity among adults fosters non-communicable diseases like cardiovascular disease, high blood pressure, and diabetes, undernutrition among children is the primary risk factor for other diseases such as acute respiratory illness, malaria, and diarrhea as well as mortalities and poor cognitive development [9, 10]. Also, poor nutritional status is associated with short- and long-term health complications including cognitive impairment and cardiometabolic diseases [11]. DBM and TBM take a toll on a country's economy with an increase in healthcare costs, decline in productivity, and downturn in development, all of which contribute to a vicious cycle of poverty and a deteriorating health system that lasts across generations [12]. Additionally, due to the cost of healthcare and the loss of human capital, undernutrition lowers Gross Domestic Product (GDP) while overweight prolongs social and economic impact on the individual with a global estimated cost of US$ 2 trillion [13].

Although studies on mother–child dyad are skewed toward one of the forms of malnutrition using small sample, such studies have indicated numerous underlying risk factors of malnutrition that include rural–urban residence, education, marital status, employment rate, environmental factors, climatic conditions, and political and economic underpinnings [14, 15]. Amidst these prevailing factors, the mothers are also affected by strenuous economic demand, single parenting, and physiological reproductive health cycles giving mothers no space to adequately replenish lost nutrients [16]. Though the nutritional status of children and mothers have improved with the implementation of supplementary feeding programs in numerous sub-Saharan African countries, the progress is incomprehensibly uneven and very slow [16, 17].

As such, identifying solutions to improve this public health issue requires much knowledge of mother–child dyads and the various forms of malnutrition. This study used data from 25 sub-Saharan African countries' Demographic and Health Surveys (DHS) to examine the prevalence and factors associated with mother–child dyads of overnutrition and undernutrition. This could add to the existing literature on mother-child dyads of overnutrition and undernutrition in sub-Saharan Africa as well as inform policymakers and programme planners to identify the risk groups and set future goals necessary to achieve the SDG 2 targets 2.1 and 2.2 by 2030.

## Methods

### Data source and design

Our study relied on data from the DHS of 25 countries in sub-Saharan Africa. We included data from the child’s record file in each of the 25 countries. We considered countries for inclusion into the study based on two criteria: countries with recent datasets spanning from 2013 to 2021 and countries with observations on all variables of interest. Evidence from the literature [18] showed that DHS has been conducted in over 90 LMICs to ascertain demographic and health-related issues, purposeful for planning, policymaking, and program management [18]. A report indicates that DHS employed a cross-sectional design [19]. Standardized and validated questionnaires were used to collect data from the respondents: men, and women. The respondents were selected for the survey using a two-stage cluster sampling technique with the detailed methodology highlighted in literature [20]. We included a weighted sample of 125,280 mother–child pairs in our study (Table 1).

**Table 1:** Description of study sample per country

Country	Year of survey	Weighted sample	Weighted %
1. Benin	2017–18	5954	4.75
2. Burundi	2016–17	5970	4.77
3. Democratic Republic of the Congo	2013–14	8043	6.42
4. Cameroon	2018	4513	3.60
5. Ethiopia	2016	5052	4.03
6. Ghana	2014	2487	1.99
7. Gambia	2019–20	3371	2.69
8. Guinea	2018	3403	2.72
9. Kenya	2014	8490	6.78
10. Liberia	2019–20	2203	1.76
11. Lesotho	2014	1365	1.09
12. Madagascar	2021	5445	4.35
13. Mali	2018	4617	3.69
14. Mauritania	2019–21	5233	4.18
15. Malawi	2015–16	7624	6.09
16. Nigeria	2018	15,032	12.00
17. Namibia	2013	2032	1.62
18. Rwanda	2019–20	3658	2.92
19. Sierra Leone	2019	4257	3.40
20. Chad	2014–15	8122	6.48
21. Togo	2013–14	2929	2.34
22.Tanzania	2015–16	4418	3.53
23. Uganda	2016	6702	5.35
24. South Africa	2016	1526	1.22
25. Zimbabwe	2015	2834	2.26
All countries	2013–2021	125,280	100.00

### Variables

#### Outcome variables

Four outcome measures were included in the study. These measures were derived from four DHS variables consisting of stunting, underweight, and wasting among children and overweight/obesity in the mother based on the mother–child pairs. We coded stunting, underweight, and wasting among children under five years based on the World Health Organization’s (WHO) Growth Reference Standard as described in previous studies [21–23]. Overweight/obesity among mothers was assessed by dividing the weight of the mother by the height squared and the resulting outcome was expressed as kilograms/meter^2^ (kg/m^2^). Based on the WHO’s standard [21] and those of previous studies [24–28] for body mass index (BMI) cut-off points: underweight, < 18.5 kg/m^2^; normal weight, 18.5–25 kg/m^2^; overweight, 25.0–29.9 kg/m^2^; and obese, ≥ 30.0 kg/m^2^, we categorized mothers whose BMI was ≥ 25.0 kg/m^2^ as overweight/obese and those whose BMI was < 25.0 kg/m^2^ as not overweight/obese. We coded each of the four Variables into “0 = normal” and “1 = ’stunted’, ‘1 = underweight’; ‘1 = wasted’, and ‘1 = overweight/obese’, respectively. Following this coding, an exclusive mother–child pair of an overweight or obese mother (OWOBM) with a stunted child (OWOBM-SC), OWOBM with an underweight child (OWOBM-UC), OWOBM with a wasted child (OWOBM-WC), and OWOBM with any form of child’s undernutrition indicators (OWOBM-SUWC) were created and used as the outcome measures [24].

#### Explanatory variables

Based on literature [22–29], we included twenty explanatory variables in our study. These variables were also available in the DHS dataset. The variables were women’s age, women’s educational level, marital status, current working status, parity, height of the women, current breastfeeding status, sex of child, age of the child, birthweight, diarrhea in the last 2 weeks, fever in the last 2 weeks, cough in the last two weeks, birth order, wealth index, source of drinking water, toilet facility, household size, place of residence, and geographical sub-region. These variables were grouped into individual and contextual level factors. Household wealth index, source of drinking water, toilet facility, household size, place of residence, and geographical sub-region were the contextual level variables with the remaining being individual level variables. Detailed categories of each variable can be found in Table 2.

**Table 2:** Distribution of OWOBM-SC, OWOBM-UC, OWOBM-WC, and OWOBM-SUWC across the explanatory variables

Variables	Weighted N (%)	OWOBM-SC	*p* value	OWOBM-UC	*p* value	OWOBM-WC	*p* value	OWOBM-SUWC	*p* value
*Child characteristics*									
Sex of child			< 0.001		< 0.001		0.025		< 0.001
Male	63,217 (50.5)	6.3		2.5		1.1		7.3	
Female	62,063 (49.5)	5.3		2.1		0.9		6.2	
Age of child			< 0.001		< 0.001		< 0.001		< 0.001
< 1	27,120 (21.6)	2.9		1.7		1.4		4.3	
1	26,153 (20.9)	6.1		2.5		1.2		7.1	
2	24,285 (19.4)	7.6		2.4		0.7		8.3	
3	24,250 (19.4)	7.0		2.4		0.7		7.7	
4	23,472 (18.7)	5.8		2.5		0.9		6.7	
Birthweight (kilograms)			< 0.001		< 0.001		< 0.001		< 0.001
2.5+	119,037 (95.0)	5.7		2.2		0.9		6.6	
Below 2.5	6243 (5.0)	8.5		4.1		1.8		9.9	
Diarrhea in the last 2 weeks			0.986		0.090		0.531		0.094
No	105,491 (84.2)	5.8		2.2		1.0		6.7	
Yes	19,789 (15.8)	5.8		2.5		1.0		6.8	
Fever in the last 2 weeks			0.005		0.318		0.271		< 0.001
No	96,024 (76.6)	6.0		2.3		1.0		6.9	
Yes	29,256 (23.4)	5.4		2.2		0.9		6.2	
Cough in the last 2 weeks			0.037		< 0.001		0.076		0.005
No	95,094 (75.9)	5.9		2.4		1.0		6.9	
Yes	30,186 (24.1)	5.5		1.9		0.9		6.3	
Currently breastfeeding			< 0.001		< 0.001		0.333		< 0.001
No	51,691 (42.3)	7.6		2.7		0.9		8.6	
Yes	73,589 (58.7)	4.6		2.0		1.0		5.5	
Birth order			< 0.001		< 0.001		0.002		< 0.001
First	26,210 (20.9)	4.0		1.4		0.8		4.8	
Second	24,361 (19.5)	5.5		2.1		0.9		6.3	
Third	20,447 (13.3)	6.0		2.2		1.0		6.9	
Fourth or more	54,262 (43.3)	6.8		2.9		1.2		7.8	
*Women’s characteristics*									
Women’s age			< 0.001		< 0.001		< 0.001		< 0.001
15–19	6877 (5.5)	2.6		0.9		0.5		3.0	
20–24	26,212 (20.9)	4.3		1.2		0.5		4.9	
25–29	34,592 (27.6)	5.4		2.2		1.0		6.3	
30–34	27,144 (21.7)	6.5		2.6		1.1		7.6	
35–39	19,276 (15.4)	7.8		3.2		1.4		9.0	
40–44	8543 (6.8)	7.9		3.1		1.4		9.2	
45–49	2636 (2.1)	6.8		3.7		1.3		7.9	
Women’s educational level			< 0.001		0.038		< 0.001		< 0.001
No education	44,260 (35.3)	5.2		2.5		1.0		6.0	
Primary	44,806 (35.8)	6.1		2.1		0.8		6.8	
Secondary	31,440 (25.1)	6.4		2.3		1.1		7.6	
Higher	4774 (3.8)	5.2		2.7		1.9		7.2	
Current working status			0.061		< 0.001		0.001		0.007
No	44,092 (35.2)	6.1		2.7		1.2		7.1	
Yes	81,188 (64.8)	5.7		2.1		0.9		6.5	
Marital status			0.211		< 0.001		0.001		0.132
Never in union	6864 (5.5)	5.2		1.7		0.8		6.2	
Married	94,377 (75.3)	5.8		2.4		1.0		6.8	
Cohabiting	16,276 (13.0)	6.0		1.8		0.7		6.7	
Widowed	1635 (1.3)	6.4		2.5		1.8		8.0	
Divorced	1972 (1.6)	7.3		3.5		0.7		8.3	
Separated	4156 (3.3)	5.6		1.5		0.5		6.2	
Parity			< 0.001		< 0.001		0.007		< 0.001
Primiparity	18,085 (14.4)	3.9		1.3		0.9		4.8	
Multiparity	63,759 (50.9)	5.6		2.1		0.9		6.5	
Grandparity	43,436 (34.7)	6.9		3.0		1.2		7.9	
Height of the women (cm)			< 0.001		< 0.001		0.195		< 0.001
Less than 145	2189 (1.8)	10.3		3.9		1.3		11.3	
145–149	8909 (7.1)	8.0		2.8		0.7		8.6	
150–154	24,563 (19.6)	7.0		2.6		0.9		7.9	
155–159	37,948 (30.3)	6.4		2.4		1.0		7.3	
160+	51,671 (41.2)	4.3		1.9		1.1		5.6	
*Household factors*									
Drinking water source			< 0.001		0.077		0.057		< 0.001
Improved	79,545 (63.5)	6.1		2.4		1.0		7.1	
Unimproved	45,735 (36.5)	5.3		2.1		0.9		6.1	
Toilet facility			< 0.001		< 0.001		0.001		< 0.001
Improved	55,504 (44.3)	6.7		2.5		1.1		7.8	
Unimproved	69,776 (55.7)	5.1		2.1		0.9		5.9	
Household size			< 0.001		< 0.001		0.007		< 0.001
Small	53,008 (42.3)	5.3		1.9		0.9		6.2	
Medium	57,417 (45.8)	6.0		2.4		1.0		7.0	
Large	14,855 (11.9)	6.7		3.2		1.2		7.9	
Wealth index			< 0.001		< 0.001		< 0.001		< 0.001
Poorest	28,204 (22.5)	4.4		1.8		0.5		4.9	
Poorer	26,803 (21.4)	5.3		2.1		0.8		6.0	
Middle	25,282 (20.2)	6.1		2.4		1.0		7.0	
Richer	23,933 (19.1)	6.7		2.4		1.3		7.9	
Richest	21,058 (16.8)	7.1		3.0		1.5		8.6	
* Community level factors*									
Place of residence			< 0.001		< 0.001		< 0.001		< 0.001
Urban	37,705 (30.1)	7.5		3.0		1.5		9.0	
Rural	87,575 (69.9)	5.1		2.0		0.7		5.8	
Geographical sub-regions			< 0.001		< 0.001		< 0.001		< 0.001
Southern	4922 (3.9)	9.8		2.9		1.6		11.3	
Central	20,678 (16.5)	4.9		1.7		0.9		5.7	
Eastern	50,194 (40.1)	4.9		1.3		0.5		5.4	
Western	49,486 (39.5)	6.8		3.4		1.5		8.2	

### Statistical analyses

We based the weighting of the dataset at the country and pooled levels before generating the results based on existing literature [30, 31]. We used percentages to summarize the proportion of OWOBM-SC, OWOBM-UC, OWOBM-WC, and OWOBM-SUWC, and presented the results on spatial maps. Next, we examined the distribution of each of OWOBM-SC, OWOBM-UC, OWOBM-WC, and OWOBM-SUWC across the explanatory variables. Pearson Chi-square test was performed to ascertain the variables that were significantly associated with each of the outcome variables at *p* < 0.05. We examined the factors associated with each of the four outcome variables using a multilevel binary logistic regression analysis. Four models were built to determine the factors associated with each of the outcome variables. In the first model (Model O), only the outcome variable was placed in it with the results indicating the variation in each of the four outcome variables attributed to the primary sampling units (PSU). Model I and II consisted of the individual and contextual level variables, respectively. Model III contained all the explanatory variables. The results were presented for each outcome variable using an adjusted odds ratio (AOR) with a 95% confidence interval (CI). Statistical significance was set at *p* < 0.05. The results in Model III were interpreted and discussed because it is the model with the least Akaike information criterion and the highest log-likelihood value. We used Stata version 17 for the analyses. We drafted this paper with reference to the Strengthening the Reporting of Observational Studies in Epidemiology (STROBE) guidelines [32].

### Ethical consideration

We did not seek ethical clearance for this study because the datasets used were freely available in the public domain. Permission to use the DHS dataset was obtained from the Monitoring and Evaluation to Assess and Use Results Demographic and Health Surveys (MEASURE DHS). We complied with the ethical guidelines guiding the use of the DHS dataset for publication.

## Results

**Fig. 1:**
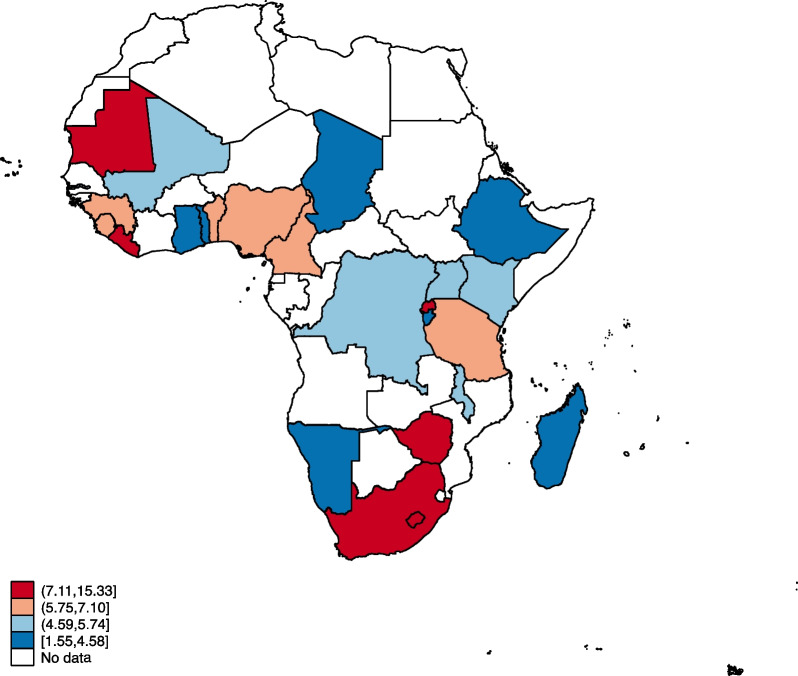
Proportion of overweight/obese mothers with stunted children in sub-Saharan Africa

**Fig. 2:**
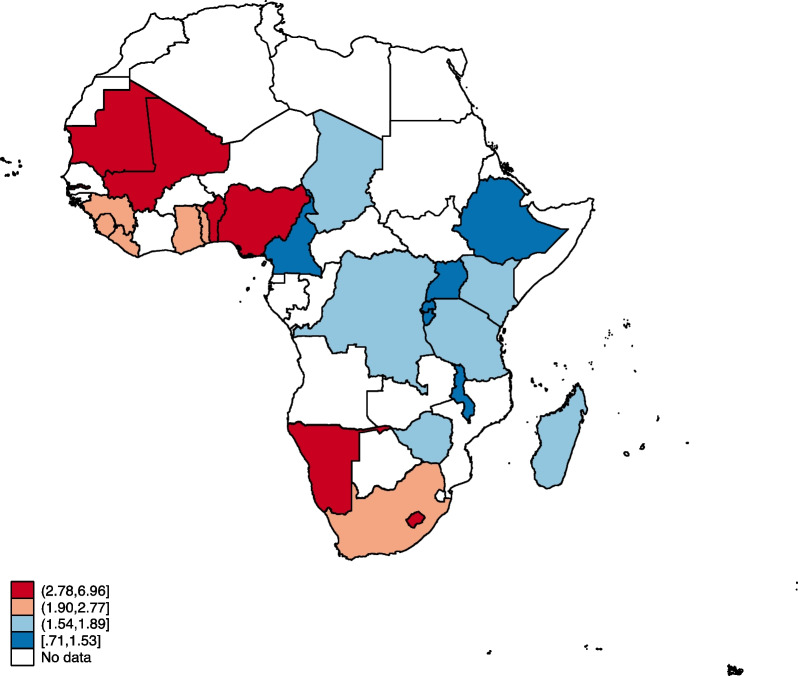
Proportion of overweight/obese mothers with underweight children in sub-Saharan Africa

**Fig. 3:**
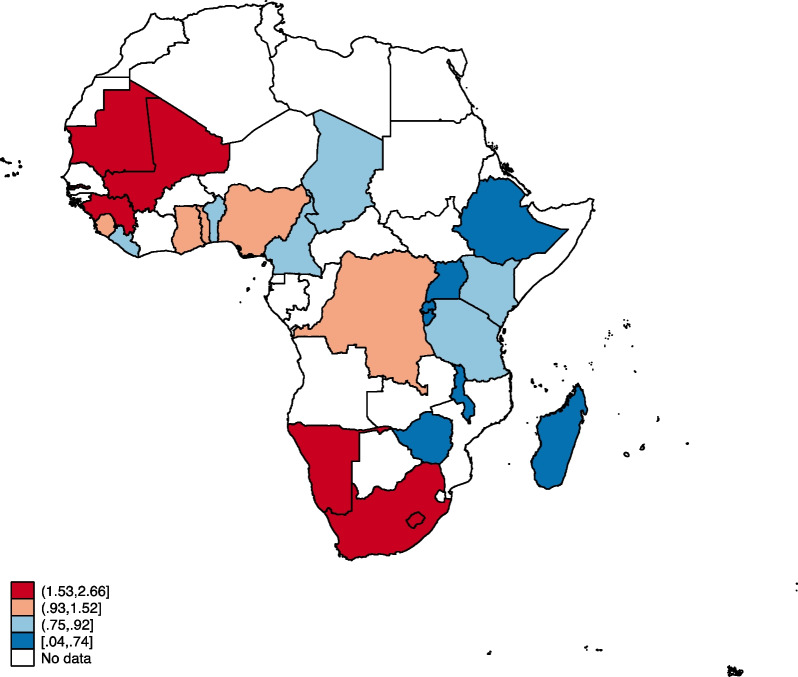
Proportion of overweight/obese mothers with wasted children in sub-Saharan Africa

**Fig. 4:**
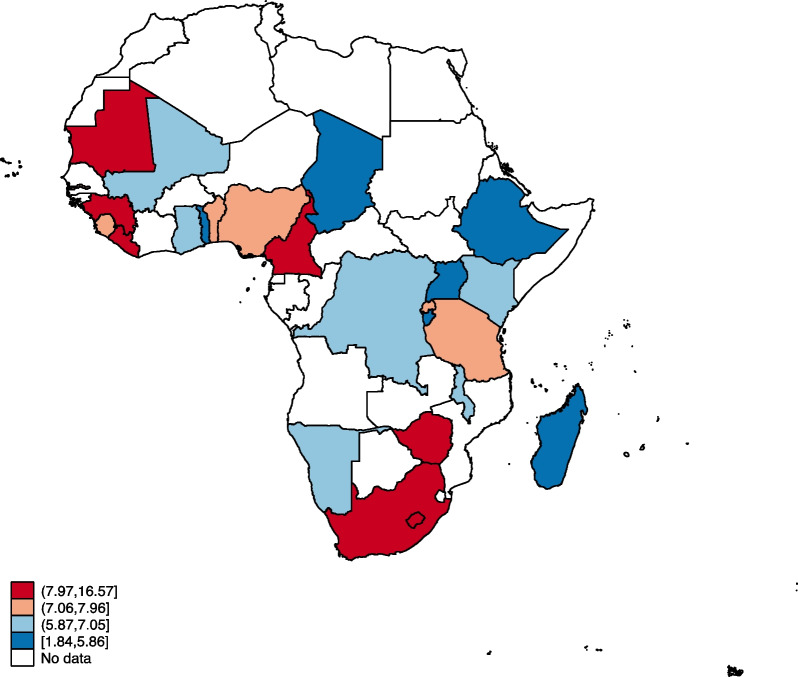
Proportion of overweight/obese mothers with undernourished children  in sub-Saharan Africa

Figures 1, 2, 3 and 4 show the distribution of OWOBM-SC, OWOBM-UC, OWOBM-WC, and OWOBM-SUWC. The countries with the highest proportions for OWOBM-SC were Cameroon, Liberia, Lesotho, Mauritania, Rwanda, South Africa, and Zimbabwe (Fig. 1). OWOBM-UC was more prevalent in Benin, Gambia, Guinea, Liberia, Lesotho, Mali, Mauritania, Nigeria, Namibia, and South Africa (Fig. 2). With OWOBM-WC, the countries with the highest proportions were Gambia, Guinea, Lesotho, Mali, Mauritania, Namibia, and South Africa (Fig. 3). Overall, the highest proportions for OWOBM-SUWC were Cameroon, Guinea, Liberia, Lesotho, Mauritania, South Africa, and Zimbabwe (Fig. 4).

### Distribution of OWOBM-SC, OWOBM-UC, OWOBM-WC, and OWOBM-SUWC across the explanatory variables

Table 2 shows the distribution of OWOBM-SC, OWOBM-UC, OWOBM-WC, and OWOBM-SUWC across the explanatory variables. There was a statistically significant association between OWOBM-SC and all the explanatory variables except for diarrhea in the last 2 weeks, current working status, and marital status at *p* < 0.05. Except for diarrhea and fever in the last 2 weeks and the source of drinking water, all the remaining variables were associated with OWOBM-UC. All but diarrhea, fever, and cough in the last 2 weeks, current breastfeeding status, height of the mother, and source of drinking water were associated with OWOBM-WC at p < 0.05. For OWOBM-SUWC, diarrhea in the last 2 weeks and marital status were the only variables with no statistically significant associations.

### Factors associated with overweight or obese mother and a child with any undernutrition indicator

Higher likelihood of OWOBM-SUWC was found among women aged 40–45 [AOR 2.20, CI 1.70, 2.85], those with primary [AOR 1.32, 95% CI 1.21, 1.44] or secondary education [AOR 1.21, 95% CI 1.09, 1.35], and divorced women [AOR 1.32, 95% CI 1.02, 1.73] compared with those aged 15–19, those with no education, and those who had never been in union, respectively. On the contrary, lower odds of OWOBM-SUWC were found among women who were currently working [AOR 0.82, 95% CI 0.76, 0.89], those currently breastfeeding [AOR 0.75, 95% CI 0.70, 0.82], and those with a height of 160 cm or more [AOR 0.29, 95% CI 0.23, 0.36] relative to those who were not working, those not breastfeeding, and those whose height was less than 145cm, respectively. The odds of OWOBM-SUWC were lower among female children [AOR 0.85, 95% CI 0.80, 0.90] compared to males. Compared to children below age 1, children of all other age groups were more likely to have OWOBM-SUWC. Other child factors that were significantly associated with OWOBM-SUWC were low birth weight [AOR 1.50, 95% CI 1.32, 1.71], having diarrhea [AOR 1.13, 95% CI 1.04, 1.24], having fever [AOR 0.91, 95% CI 0.83, 0.99], and higher birth order [AOR 1.37, 95% CI 1.13, 1.66]. Belonging to the richest wealth index [AOR 1.49, 95% CI 1.28, 1.72] was associated with higher likelihood of having OWOBM-SUWC. Using unimproved toilet facilities [AOR 0.90, 95% CI 0.83, 0.98] and rural residency [AOR 0.79, 95% CI 0.71, 0.87] were less likely to be associated with OWOBM-SUWC. Children from the Central [AOR 0.55, 95% CI 0.46, 0.65], Eastern [AOR 0.44, 95% CI 0.38, 0.52], and Western [AOR 0.76, 95% CI 0.65, 0.89] sub-Saharan Africa had significantly lower odds of OWOBM-SUWC compared to those from the Southern sub-region (see Table 3). Other factors such as maternal age, child’s age, level of education, employment status, wealth index, and breastfeeding status were significantly associated with OWOBM-SC, OWOBM-UC, OWOBM-WC (Additional file 1: Tables S1–3).

**Table 3:** Multilevel analysis results of factors associated with overweight or obese mother and a child with any undernutrition indicator

Variable	Model O	Model I AOR [95% CI]	Model II AOR [95% CI]	Model III AOR [95% CI]
*Fixed effect model*				
Women’s age				
15–19		1.00		1.00
20–24		1.43*** [1.16, 1.75]		1.40** [1.14, 1.72]
25–29		1.76*** [1.42, 2.18]		1.62*** [1.30, 2.01]
30–34		2.10*** [1.66, 2.64]		1.85*** [1.47, 2.34]
35–39		2.51*** [1.96, 3.21]		2.17*** [1.70, 2.79]
40–44		2.51*** [1.95, 3.24]		2.20*** [1.70, 2.85]
45–49		2.12*** [1.56, 2.88]		1.83*** [1.34, 2.51]
Women’s educational level				
No education		1.00		1.00
Primary		1.21*** [1.11, 1.31]		1.32*** [1.21, 1.44]
Secondary		1.46*** [1.32, 1.61]		1.21*** [1.09, 1.35]
Higher		1.32** [1.09, 1.60]		1.01 [0.82, 1.24]
Marital status				
Never in union		1.00		1.00
Married		0.92 [0.78, 1.09]		1.06 [0.89, 1.26]
Cohabiting		0.93 [0.77, 1.12]		1.12 [0.93, 1.36]
Widowed		0.91 [0.68, 1.22]		1.11 [0.82, 1.48]
Divorced		1.10 [0.84, 1.44]		1.32* [1.02, 1.73]
Separated		0.82 [0.65, 1.04]		1.06 [0.84, 1.34]
Current working status				
No		1.00		1.00
Yes		0.79*** [0.74, 0.85]		0.82*** [0.76, 0.89]
Parity				
Primiparity		1.00		1.00
Multiparity		1.08 [0.89, 1.30]		1.04 [0.86, 1.26]
Grandparity		1.16 [0.93, 1.46]		1.16 [0.93, 1.46]
Height of the women (cm)				
Less than 145		1.00		1.00
145–149		0.67*** [0.54, 0.84]		0.63*** [0.51, 0.79]
150–154		0.60*** [0.49, 0.74]		0.53*** [0.43, 0.66]
155–159		0.54*** [0.44, 0.66]		0.46*** [0.37, 0.56]
160+		0.35*** [0.29, 0.43]		0.29*** [0.23, 0.36]
Currently breastfeeding				
No		1.00		1.00
Yes		0.70*** [0.65, 0.76]		0.75*** [0.70, 0.82]
Sex of child				
Male		1.00		1.00
Female		0.84*** [0.80, 0.89]		0.85*** [0.80, 0.90]
Age of child				
< 1		1.00		1.00
1		1.57*** [1.41, 1.75]		1.60*** [1.44, 1.79]
2		1.59*** [1.43, 1.77]		1.68*** [1.51, 1.87]
3		1.45*** [1.30, 1.62]		1.55*** [1.39, 1.73]
4		1.23*** [1.09, 1.37]		1.31*** [1.16, 1.47]
Birthweight (kilograms)				
2.5+		1.00		1.00
Below 2.5		1.54*** [1.36, 1.76]		1.50*** [1.32, 1.71]
Diarrhea in the last 2 weeks				
No		1.00		1.00
Yes		1.12* [1.03, 1.23]		1.13** [1.04, 1.24]
Fever in the last 2 weeks				
No		1.00		1.00
Yes		0.90* [0.83, 0.98]		0.91* [0.83, 0.99]
Cough in the last 2 weeks				
No		1.00		1.00
Yes		0.90** [0.84, 0.97]		0.96 [0.89, 1.04]
Birth order				
First		1.00		1.00
Second		1.16 [1.00, 1.36]		1.21* [1.04, 1.41]
Third		1.24* [1.05, 1.47]		1.32** [1.11, 1.57]
Fourth or more		1.25* [1.04, 1.51]		1.37** [1.13, 1.66]
Wealth index				
Poorest			1.00	1.00
Poorer			1.19** [1.07, 1.32]	1.18** [1.06, 1.32]
Middle			1.33*** [1.19, 1.48]	1.31*** [1.18, 1.46]
Richer			1.39*** [1.23, 1.57]	1.40*** [1.23, 1.58]
Richest			1.41*** [1.22, 1.62]	1.49*** [1.28, 1.72]
Drinking water source				
Improved			1.00	1.00
Unimproved			0.95 [0.88, 1.03]	0.95 [0.88, 1.03]
Toilet facility				
Improved			1.00	1.00
Unimproved			0.90** [0.83, 0.97]	0.90** [0.83, 0.98]
Household size				
Small			1.00	1.00
Medium			1.17*** [1.09, 1.25]	0.99 [0.91, 1.07]
Large			1.20*** [1.08, 1.33]	1.10 [0.99, 1.23]
Place of residence				
Urban			1.00	1.00
Rural			0.77*** [0.70, 0.85]	0.79*** [0.71, 0.87]
Geographical sub-region				
Southern			1.00	1.00
Central			0.49*** [0.41, 0.58]	0.55*** [0.46, 0.65]
Eastern			0.47*** [0.41, 0.55]	0.44*** [0.38, 0.52]
Western			0.68*** [0.59, 0.78]	0.76*** [0.65, 0.89]
*Random effect model*				
PSU variance (95% CI)	0.529 [0.456, 0.615]	0.524 [0.448, 0.612]	0.506 [0.431, 0.593]	0.517 [0.438, 0.611]
ICC	0.138	0.137	0.133	0.136
Wald Chi-square	Reference	1182.55 (< 0.001)	442.21 (< 0.001)	1505.79 (< 0.001)
Model fitness				
Log-likelihood	− 68,942.48	− 66,668.802	− 67,959.413	− 65,706.517
AIC	137,889	133,409.6	135,946.8	131,509
BIC	137,908.4	133,760.2	136,083.2	131,976.5
N	125,280	125,280	125,280	125,280
Number of clusters	1608	1608	1608	1608

aOR, adjusted odds ratios; CI, confidence interval; *p < 0.05, **p < 0.01, ***p < 0.001; 1.00 = Reference category; PSU, primary sampling unit; ICC, intra-class correlation; AIC, Akaike information criterion; BIC, Bayesian information criterion

## Discussion

In this study, we examined mother–child dyads of overnutrition and undernutrition in sub-Saharan Africa. The results indicate that there are maternal, child, and contextual factors associated with this public health phenomenon. Results from the spatial analysis identified Lesotho and South Africa to be consistent hotspots for OWOBM-SC, OWOBM-WC, and OWOBM-SUWC. Prior literature has shown that in 2007, a simultaneous crop failure occurred in Lesotho and South Africa, which happens to be the only trading partner of Lesotho [33]. This resulted in a significant period of food insecurity in Lesotho [33]. Therefore, the aftermath of the period of high food insecurity may persist and latently influence mother–child dyads of overnutrition and undernutrition. The effect of climate change on food availability and quality and limited education on adequate nutrition could have contributed to the occurrence of undernutrition among children and overweight/obese mothers. Nevertheless, these hotspots signify areas in sub-Saharan Africa where efforts to combat malnutrition should focus, considering the specific combination of maternal overweight/obesity and child undernutrition observed in each location.

Consistent with Keats et al.’s [34] study which reported that maternal characteristics play a quintessential role in determining the nutritional status of children, we found that the mother’s height has a strong inverse association with OWOBM-SUWC. That is, children born to tall overweight or obese mothers were less likely to be stunted, wasted, or underweight. Similar findings have been reported in a study that was conducted in India [24] where a mother’s short stature was associated with a higher risk of OWOBM-SUWC. The result also aligns with that of Mahmudiono et al. [35] who asserted that maternal height significantly predicts a DBM. This association may be linked to the inadequate anatomical and metabolic systems of mothers with short stature, which leads to intrauterine growth restriction [36]. Hence, the child is also likely to experience OWOBM-SUWC because of an intergeneration transfer of malnutrition [24]. Özaltin et al. [37] also explain this association from the perspective that “for mothers, limited nutrient supply at the cellular level during their development may lead to maintenance of basic metabolic functions taking precedence and resources being diverted away from growth, resulting in growth retardation and shortened stature”.

There was a positive association between educational attainment, OWOBM-SC and OWOBM-UC but not for OWOBM-WC. Children born to overweight or obese mothers who had formal education were more likely to be stunted or underweight but not wasted. The result is inconsistent with Kumar and Mohanty's [24] study that found educated mothers to be less likely to have a child with any undernutrition indicators. Although the direction of the association was unexpected, it suggests that even with formal education, overweight or obese mothers may face challenges in providing optimal nutrition to their children, leading to stunting or underweight. The demands and stress associated with professional responsibilities may affect the time and attention available for optimal childcare practices. The juggling act between career commitments and childcare responsibilities might inadvertently compromise the ability of educated mothers to ensure their children receive the necessary nutritional care, leading to an increased likelihood of stunting or being underweight. Moreover, the nature of formal employment can contribute to heightened stress levels among educated mothers. Occupational stressors, coupled with the demands of maintaining a healthy work-life balance, may impact maternal well-being and, consequently, the ability to provide adequate nutrition to their children. This is particularly relevant as stress has been linked to disruptions in dietary routines and nutritional choices. Additionally, societal expectations and pressures on educated mothers to excel both professionally and in their caregiving roles may create a challenging environment. The pursuit of perfection in multiple domains may inadvertently divert attention from maintaining optimal nutrition for their children. Perhaps that can explain why educated mothers, who are expected to have better knowledge of dietary practices and access to resources [38], were more likely to have children who were stunted or underweight.

Contrary to an earlier study conducted in India [24], current working status was significantly associated with the nutritional status of children born to overweight or obese mothers. The observed inverse association can be explained from the perspective that working mothers, irrespective of being overweight or obese, may have better access to healthcare services, either through their employer-provided health insurance or their ability to afford private healthcare [39]. This can lead to improved prenatal care during pregnancy, access to nutrition education, and regular check-ups for their children, which can positively affect their nutritional status. In addition, being employed can enhance a woman's sense of empowerment and agency [40]. This increased empowerment may result in greater decision-making power within the household, including choices related to nutrition and healthcare for their children. When women can make informed decisions and have control over resources, it can contribute to better outcomes for their children's nutritional status.

We found the odds of OWOBM-SUWC to be lower in situations where the overweight or obese mother was currently breastfeeding. This affirms prior studies [36, 41] that have established a strong negative association between breastfeeding practices and nutritional health outcomes among children under five. Thus, underscoring a need for sub-Saharan African countries to emphasize and improve breastfeeding practices among women. Our study also shows that the likelihood of OWOBM-SUWC occurring increases with maternal age – a finding that is surprising as it is expected that women of a younger age would have their children being more likely to be stunted, wasted, or underweight due to the stigma of birth at a young age, lack of resources and naivety about child dietary practices [42, 43]. It is unclear the reasons for this association. Further studies would be required to fully comprehend the extent and significance of older maternal age in explaining mother–child dyads of over- and undernutrition in SSA.

We found the odds of OWOBM-SUWC to be significantly low among female children compared to males. This is inconsistent when compared to prior studies that have found no significant association [24, 44] between sex and mother–child dyads of overnutrition and undernutrition or a higher risk among females compared to males [45]. The higher risk of stunting and underweight among males can be explained from an evolutionary perspective, specifically through the lens of selective male mortality. This theory suggests that differences in the genetic composition between males and females contribute to the increased vulnerability of males to stunting and being underweight [46, 47]. In addition, our study shows that older children were more likely to have OWOBM-SUWC, which is consistent with some studies [46, 48]. Previous research [49] has demonstrated that childhood anemia, faltering growth, stunting, wasting, and underweight are more prevalent in children beyond infancy. This finding helps to explain why the odds of OWOBM-SUWC are higher among older children compared to those who are still in their first year of life.

Low birth weight children and those of higher birth order were at higher risk of stunting, wasting, and underweight if their mother was overweight or obese. This aligns with a study conducted in India [24, 48]. Higher birth order may be an indication of a cumulative burden on the mother's health and resources, which can impact the nutritional status of subsequent children. 

The observed sub-regional differences are similar to the findings reported by Ahinkorah et al. [46]. It reflects the differences in policies and commitments related to addressing malnutrition. For instance, a study [50] revealed that among the examined regions, three out of 16 countries in Western Africa, two out of five in Southern Africa, four out of nine in Central Africa, and seven out of 18 in Eastern Africa have implemented multisectoral comprehensive nutrition plans. The policy variations across sub-regions indicate the diverse approaches taken to tackle malnutrition and suggest that regional efforts toward addressing this issue may differ in their comprehensiveness and effectiveness.

## Strengths and limitations

The major strength of this study lies in the use of recent DHS dataset to examine mother-child dyads of overnutrition and undernutrition in sub-Saharan Africa. However, the cross-sectional nature of the DHS does not allow us to make causal inferences. Also, it is uncertain whether the mothers’ overweight or obese status occurred before the health outcome of the child, or vice versa. The study excluded physical activity, dietary intakes, caregiving approaches, and cultural aspects as they were not available in the DHS dataset. Another noteworthy limitation of this study was the use of BMI as an indicator of overweight/obesity. This is because BMI does not distinguish between lean body mass and fat mass. This lack of specificity can be particularly relevant in the context of maternal health, as women with higher muscle mass may be categorized as overweight or obese based on BMI alone, despite having a lower percentage of body fat. Furthermore, BMI does not account for the distribution of body fat, and different individuals may have varying fat distribution patterns. Two individuals with the same BMI may have different proportions of visceral and subcutaneous fat, which have distinct metabolic implications. This limitation is especially pertinent given the growing recognition of the significance of visceral adiposity in influencing health outcomes.

## Conclusion

A combination of child, maternal, and contextual-related factors explains mother–child dyads of overnutrition and undernutrition in sub-Saharan Africa. Addressing this situation requires policies and interventions that empower women through education and economic engagement. The observed sub-regional differences in policies and commitments related to addressing malnutrition suggest the need for comprehensive and coordinated efforts. Governments and stakeholders should strive to implement and strengthen multisectoral comprehensive nutrition plans across sub-regions. Sharing best practices and lessons learned can help improve the effectiveness and comprehensiveness of nutrition interventions and contribute to reducing the prevalence of malnutrition.

### Supplementary Information

**Additional file 1. Table S1.** Multilevel analysis results of factors associated with overweight or obese mother and stunted child. **Table S2.** Multilevel analysis results of factors associated with overweight or obese mother and underweight child. **Table S3.** Multilevel analysis results of factors associated with overweight or obese mother and wasted child.

## Data Availability

Data for this study were sourced from Demographic and Health surveys (DHS) and available here: http://dhsprogram.com/data/available-datasets.cfm.

## Abbreviations

AIC: Akaike Information Criterion
AOR: Adjusted odds ratio
CI: Confidence interval
DHS: Demographic and Health Survey
ICC: Intra-class correlation
OWOBM-SC: Overweight or obese mother with a stunted child
OWOBM-SUWC: Overweight or obese mother with a stunted, underweight, and wasted child
OWOBM-UC: Overweight or obese mother with an underweight child
OWOBM-WC: Overweight or obese mother with a wasted child
OWOBM: Overweight or obese mother
PSU: Primary sampling unit
