# Supplementary material for: Mother–child dyads of overnutrition and undernutrition in sub-Saharan Africa
Source: J Health Popul Nutr. 2024 Jan 2;43:1. doi: 10.1186/s41043-023-00479-y (PMC10759505; doi:10.1186/s41043-023-00479-y)
Supplement: Supplementary file 1 — Additional file 1. Table S1. Multilevel analysis results of factors associated with overweight or obese mother and stunted child. Table S2. Multilevel analysis results of factors associated with overweight or obese mother and underweight child. Table S3. Multilevel analysis results of factors associated with overweight or obese mother and wasted child. [file 41043_2023_479_MOESM1_ESM.docx]

**Supplementary Tables**

**Table S1. Multilevel analysis results of factors associated with overweight or obese mother and stunted child**

| **Variable** | **Model O** | **Model I**  **AOR [95% CI]** | **Model II**  **AOR [95% CI]** | **Model III**  **AOR [95% CI]** |
| --- | --- | --- | --- | --- |
| **Fixed effect model** |  |  |  |  |
| **Women’s age** |  |  |  |  |
| 15-19 |  | 1.00 |  | 1.00 |
| 20-24 |  | 1.34^**^ [1.08, 1.67] |  | 1.32^*^ [1.06, 1.64] |
| 25-29 |  | 1.56^***^ [1.24, 1.96] |  | 1.44^**^ [1.15, 1.81] |
| 30-34 |  | 1.80^***^ [1.41, 2.31] |  | 1.61^***^ [1.26, 2.07] |
| 35-39 |  | 2.17^***^ [1.66, 2.82] |  | 1.90^***^ [1.45, 2.48] |
| 40-44 |  | 2.14^***^ [1.64, 2.81] |  | 1.90^***^ [1.44, 2.50] |
| 45-49 |  | 1.79^***^ [1.29, 2.48] |  | 1.57^**^ [1.13, 2.19] |
| **Women’s educational level** |  |  |  |  |
| No education |  | 1.00 |  | 1.00 |
| Primary |  | 1.24^***^ [1.14, 1.36] |  | 1.34^***^ [1.22, 1.47] |
| Secondary |  | 1.44^***^ [1.29, 1.60] |  | 1.21^**^ [1.08, 1.35] |
| Higher |  | 1.16 [0.94, 1.43] |  | 0.90 [0.72, 1.13] |
| **Marital status** |  |  |  |  |
| Never in union |  | 1.00 |  | 1.00 |
| Married |  | 0.91 [0.76, 1.09] |  | 1.07 [0.88, 1.29] |
| Cohabiting |  | 0.95 [0.78, 1.17] |  | 1.17 [0.95 , 1.44] |
| Widowed |  | 0.80 [0.59, 1.09] |  | 0.98 [0.72, 1.35] |
| Divorced |  | 1.09 [0.82, 1.46] |  | 1.33^*^ [1.00, 1.78] |
| Separated |  | 0.84 [0.66, 1.08] |  | 1.09 [0.85, 1.39] |
| **Current working status** |  |  |  |  |
| No |  | 1.00 |  | 1.00 |
| Yes |  | 0.80^***^ [0.74, 0.86] |  | 0.83^***^ [0.77, 0.90] |
| **Parity** |  |  |  |  |
| Primiparity |  | 1.00 |  | 1.00 |
| Multiparity |  | 1.11 [0.91, 1.36] |  | 1.08 [0.88, 1.32] |
| Grandparity |  | 1.22 [0.96, 1.54] |  | 1.20 [0.95, 1.53] |
| **Height of the women (cm)** |  |  |  |  |
| Less than 145 |  | 1.00 |  | 1.00 |
| 145-149 |  | 0.70^**^ [0.56, 0.87] |  | 0.66^***^ [0.52, 0.83] |
| 150-154 |  | 0.59^***^ [0.48, 0.73] |  | 0.53^***^ [0.43, 0.66] |
| 155-159 |  | 0.52^***^ [0.42, 0.64] |  | 0.45^***^ [0.36, 0.56] |
| 160+ |  | 0.31^***^ [0.25, 0.39] |  | 0.26^***^ [0.21, 0.33] |
| **Currently breastfeeding** |  |  |  |  |
| No |  | 1.00 |  | 1.00 |
| Yes |  | 0.70^***^ [0.64, 0.76] |  | 0.74^***^ [0.68, 0.81] |
| **Sex of child** |  |  |  |  |
| Male |  | 1.00 |  | 1.00 |
| Female |  | 0.82^***^ [0.77, 0.87] |  | 0.82^***^ [0.77, 0.88] |
| **Age of child** |  |  |  |  |
| 0 |  | 1.00 |  | 1.00 |
| 1 |  | 2.04^***^ [1.81, 2.31] |  | 2.08^***^ [1.84, 2.36] |
| 2 |  | 2.23^***^ [1.97, 2.52] |  | 2.34^***^ [2.07, 2.65] |
| 3 |  | 2.02^***^ [1.78, 2.28] |  | 2.14^***^ [1.89, 2.42] |
| 4 |  | 1.60^***^ [1.40, 1.83] |  | 1.70^***^ [1.48, 1.94] |
| **Birthweight (kilograms)** |  |  |  |  |
| 2.5+ |  | 1.00 |  | 1.00 |
| Below 2.5 |  | 1.53^***^ [1.33, 1.77] |  | 1.49^***^ [1.29, 1.72] |
| **Diarrhoea in the last 2 weeks** |  |  |  |  |
| No |  | 1.00 |  | 1.00 |
| Yes |  | 1.13^*^ [1.02, 1.24] |  | 1.13^**^ [1.03, 1.25] |
| **Fever in the last 2 weeks** |  |  |  |  |
| No |  | 1.00 |  | 1.00 |
| Yes |  | 0.89^*^ [0.82, 0.98] |  | 0.90^*^ [0.82, 0.99] |
| **Cough in the last 2 weeks** |  |  |  |  |
| No |  | 1.00 |  | 1.00 |
| Yes |  | 0.92^*^ [0.85, 1.00] |  | 0.97 [0.90, 1.06] |
| **Birth order** |  |  |  |  |
| First |  | 1.00 |  | 1.00 |
| Second |  | 1.22^*^ [1.04 ,1.44] |  | 1.27^**^ [1.08, 1.49] |
| Third |  | 1.30^**^ [1.09, 1.55] |  | 1.37^***^ [1.15, 1.64] |
| Fourth or more |  | 1.33^**^ [1.09, 1.62] |  | 1.43^***^ [1.18, 1.74] |
| **Wealth index** |  |  |  |  |
| Poorest |  |  | 1.00 | 1.00 |
| Poorer |  |  | 1.17^**^ [1.05, 1.31] | 1.16^*^ [1.04, 1.30] |
| Middle |  |  | 1.28^***^ [1.14, 1.44] | 1.27^***^ [1.13, 1.42] |
| Richer |  |  | 1.30^***^ [1.15, 1.47] | 1.31^***^ [1.15, 1.48] |
| Richest |  |  | 1.27^**^ [1.10, 1.47] | 1.38^***^ [1.18, 1.62] |
| **Drinking water source** |  |  |  |  |
| Improved |  |  | 1.00 | 1.00 |
| Unimproved |  |  | 0.95 [0.87, 1.03] | 0.95 [0.88, 1.04] |
| **Toilet facility** |  |  |  |  |
| Improved |  |  | 1.00 | 1.00 |
| Unimproved |  |  | 0.88^**^ [0.81, 0.96] | 0.88^**^ [0.81, 0.96] |
| **Household size** |  |  |  |  |
| Small |  |  | 1.00 | 1.00 |
| Medium |  |  | 1.18^***^ [1.10, 1.27] | 1.00 [0.92, 1.09] |
| Large |  |  | 1.23^***^ [1.10, 1.37] | 1.14^*^ [1.02, 1.28] |
| **Place of residence** |  |  |  |  |
| Urban |  |  | 1.00 | 1.00 |
| Rural |  |  | 0.79^***^ [0.71, 0.87] | 0.80^***^ [0.72, 0.88] |
| **Geographical subregions** |  |  |  |  |
| Southern |  |  | 1.00 | 1.00 |
| Central |  |  | 0.50^***^ [0.42, 0.60] | 0.55^***^ [0.46, 0.67] |
| Eastern |  |  | 0.50^***^ [0.43, 0.59] | 0.46^***^ [0.38, 0.54] |
| Western |  |  | 0.65^***^ [0.56, 0.76] | 0.73^***^ [0.61, 0.86] |
| **Random effect model** |  |  |  |  |
| PSU variance (95% CI) | 0.565 [0.486, 0.656] | 0.576 [0.493, 0.673] | 0.549 [0.470, 0.642] | 0.571 [0.485, 0.673] |
| ICC | 0.146 | 0.149 | 0.143 | 0.148 |
| Wald chi-square | Reference | 1232.23 (<0.001) | 287.44 (<0.001) | 1484.54 (<0.001) |
| **Model fitness** |  |  |  |  |
| Log-likelihood | -61891.753 | -59363.842 | -61231.774 | -58669.554 |
| AIC | 123787.5 | 118799.7 | 122491.5 | 117435.1 |
| BIC | 123807 | 119150.3 | 122627.9 | 117902.5 |
| N | 125280 | 125280 | 125280 | 125280 |
| Number of clusters | 1608 | 1608 | 1608 | 1608 |

AOR= adjusted odds ratios; CI= Confidence Interval; ^*^ *p* < 0.05, ^**^ *p* < 0.01, ^***^ *p* < 0.001; 1.00 = Reference category; PSU=Primary Sampling Unit; ICC = Intra-Class Correlation; AIC = Akaike’s Information Criterion

**Table S2. Multilevel analysis results of factors associated with overweight or obese mother and underweight child**

| **Variable** | **Model O** | **Model I**  **AOR [95% CI]** | **Model II**  **AOR [95% CI]** | **Model III**  **AOR [95% CI]** |
| --- | --- | --- | --- | --- |
| **Fixed effect model** |  |  |  |  |
| **Women’s age** |  |  |  |  |
| 15-19 |  | 1.00 |  | 1.00 |
| 20-24 |  | 1.20 [0.85, 1.69] |  | 1.23 [0.87, 1.74] |
| 25-29 |  | 1.98^***^ [1.41, 2.80] |  | 1.93^***^ [1.37, 2.72] |
| 30-34 |  | 2.22^***^ [1.51, 3.26] |  | 2.10^***^ [1.43, 3.09] |
| 35-39 |  | 2.61^***^ [1.73, 3.94] |  | 2.44^***^ [1.61, 3.69] |
| 40-44 |  | 2.45^***^ [1.63, 3.69] |  | 2.31^***^ [1.53, 3.49] |
| 45-49 |  | 2.88^***^ [1.78, 4.68] |  | 2.60^***^ [1.59, 4.24] |
| **Women’s educational level** |  |  |  |  |
| No education |  | 1.00 |  | 1.00 |
| Primary |  | 0.94 [0.83, 1.07] |  | 1.20^**^ [1.05, 1.38] |
| Secondary |  | 1.13 [0.97, 1.30] |  | 1.04 [0.89, 1.22] |
| Higher |  | 1.30 [0.96, 1.76] |  | 1.11 [0.81, 1.51] |
| **Marital status** |  |  |  |  |
| Never in union |  | 1.00 |  | 1.00 |
| Married |  | 1.09 [0.82, 1.46] |  | 1.16 [0.86, 1.56] |
| Cohabiting |  | 0.87 [0.63, 1.19] |  | 1.06 [0.77, 1.46] |
| Widowed |  | 0.95 [0.59, 1.53] |  | 1.11 [0.69, 1.81] |
| Divorced |  | 1.63^*^ [1.06, 2.51] |  | 1.87^**^ [1.22, 2.88] |
| Separated |  | 0.73 [0.48, 1.11] |  | 0.97 [0.64, 1.47] |
| **Current working status** |  |  |  |  |
| No |  | 1.00 |  | 1.00 |
| Yes |  | 0.68^***^ [0.61, 0.76] |  | 0.69^***^ [0.62, 0.77] |
| **Parity** |  |  |  |  |
| Primiparity |  | 1.00 |  | 1.00 |
| Multiparity |  | 1.12 [0.81, 1.54] |  | 1.04 [0.75, 1.44] |
| Grandparity |  | 1.28 [0.88, 1.87] |  | 1.20 [0.82, 1.76] |
| **Height of the women (cm)** |  |  |  |  |
| Less than 145 |  | 1.00 |  | 1.00 |
| 145-149 |  | 0.66^*^ [0.47, 0.92] |  | 0.60^**^ [0.43, 0.84] |
| 150-154 |  | 0.58^***^ [0.43, 0.77] |  | 0.49^***^ [0.36, 0.66] |
| 155-159 |  | 0.51^***^ [0.38, 0.68] |  | 0.40^***^ [0.30, 0.55] |
| 160+ |  | 0.38^***^ [0.28, 0.51] |  | 0.28^***^ [0.21, 0.39] |
| **Currently breastfeeding** |  |  |  |  |
| No |  | 1.00 |  | 1.00 |
| Yes |  | 0.82^**^ [0.72, 0.92] |  | 0.88^*^ [0.77, 0.99] |
| **Sex of child** |  |  |  |  |
| Male |  | 1.00 |  | 1.00 |
| Female |  | 0.83^***^ [0.76, 0.92] |  | 0.84^***^ [0.77, 0.93] |
| **Age of child** |  |  |  |  |
| 0 |  | 1.00 |  | 1.00 |
| 1 |  | 1.39^***^ [1.18, 1.65] |  | 1.43^***^ [1.21, 1.69] |
| 2 |  | 1.24^*^ [1.05, 1.47] |  | 1.32^**^ [1.11, 1.56] |
| 3 |  | 1.21^*^ [1.01, 1.44] |  | 1.32^**^ [1.11, 1.57] |
| 4 |  | 1.19 [1.00, 1.43] |  | 1.29^**^ [1.08, 1.55] |
| **Birthweight (kilograms)** |  |  |  |  |
| 2.5+ |  | 1.00 |  | 1.00 |
| Below 2.5 |  | 2.06^***^ [1.72, 2.46] |  | 2.03^***^ [1.70, 2.44] |
| **Diarrhoea in the last 2 weeks** |  |  |  |  |
| No |  | 1.00 |  | 1.00 |
| Yes |  | 1.26^**^ [1.09, 1.45] |  | 1.28^***^ [1.11, 1.48] |
| **Fever in the last 2 weeks** |  |  |  |  |
| No |  | 1.00 |  | 1.00 |
| Yes |  | 1.03 [0.91, 1.17] |  | 1.03 [0.91, 1.18] |
| **Cough in the last 2 weeks** |  |  |  |  |
| No |  | 1.00 |  | 1.00 |
| Yes |  | 0.77^***^ [0.68, 0.89] |  | 0.88 [0.76, 1.01] |
| **Birth order** |  |  |  |  |
| First |  | 1.00 |  | 1.00 |
| Second |  | 1.23 [0.94, 1.62] |  | 1.32^*^ [1.01, 1.74] |
| Third |  | 1.18 [0.88, 1.59] |  | 1.28 [0.96, 1.72] |
| Fourth or more |  | 1.25 [0.90, 1.74] |  | 1.39 [1.00, 1.93] |
| **Wealth index** |  |  |  |  |
| Poorest |  |  | 1.00 | 1.00 |
| Poorer |  |  | 1.14 [0.96, 1.36] | 1.16 [0.97, 1.38] |
| Middle |  |  | 1.23^*^ [1.04, 1.45] | 1.25^**^ [1.06, 1.48] |
| Richer |  |  | 1.16 [0.95, 1.42] | 1.21 [0.99, 1.49] |
| Richest |  |  | 1.40^**^ [1.13, 1.73] | 1.53^***^ [1.22, 1.91] |
| **Drinking water source** |  |  |  |  |
| Improved |  |  | 1.00 | 1.00 |
| Unimproved |  |  | 0.96 [0.85, 1.09] | 0.96 [0.85, 1.09] |
| **Toilet facility** |  |  |  |  |
| Improved |  |  | 1.00 | 1.00 |
| Unimproved |  |  | 0.94 [0.84, 1.07] | 0.94 [0.83, 1.06] |
| **Household size** |  |  |  |  |
| Small |  |  | 1.00 | 1.00 |
| Medium |  |  | 1.27^***^ [1.13, 1.44] | 1.02 [0.90, 1.15] |
| Large |  |  | 1.47^***^ [1.25, 1.73] | 1.24^*^ [1.05, 1.46] |
| **Place of residence** |  |  |  |  |
| Urban |  |  | 1.00 | 1.00 |
| Rural |  |  | 0.85^*^ [0.73, 0.98] | 0.86^*^ [0.74, 1.00] |
| **Geographical subregions** |  |  |  |  |
| Southern |  |  | 1.00 | 1.00 |
| Central |  |  | 0.61^***^ [0.46, 0.79] | 0.64^**^ [0.48, 0.84] |
| Eastern |  |  | 0.48^***^ [0.37, 0.62] | 0.43^***^ [0.33, 0.57] |
| Western |  |  | 1.13 [0.88, 1.44] | 1.16 [0.89, 1.51] |
| **Random effect model** |  |  |  |  |
| PSU variance (95% CI) | 1.036 [0.888, 1.209] | 1.009 [0.863, 1.180] | 0.990 [0.845, 1.160] | 0.970 [0.824, 1.142] |
| ICC | 0.239 | 0.235 | 0.231 | 0.228 |
| Wald chi-square | Reference | 502.65 (<0.001) | 322.31 (<0.001) | 763.63 (<0.001) |
| **Model fitness** |  |  |  |  |
| Log-likelihood | -30032.223 | -29136.944 | -29370.531 | -28502.594 |
| AIC | 60068.45 | 58345.89 | 58769.06 | 57101.19 |
| BIC | 60087.92 | 58696.47 | 58905.4 | 57568.63 |
| N | 125280 | 125280 | 125280 | 125280 |
| Number of clusters | 1608 | 1608 | 1608 | 1608 |

AOR= adjusted odds ratios; CI= Confidence Interval; ^*^ *p* < 0.05, ^**^ *p* < 0.01, ^***^ *p* < 0.001; 1.00 = Reference category; PSU=Primary Sampling Unit; ICC = Intra-Class Correlation; AIC = Akaike’s Information Criterion

**Table S3. Multilevel analysis results of factors associated with overweight or obese mother and wasted child**

| **Variable** | **Model O** | **Model I**  **AOR [95% CI]** | **Model II**  **AOR [95% CI]** | **Model III**  **AOR [95% CI]** |
| --- | --- | --- | --- | --- |
| **Fixed effect model** |  |  |  |  |
| **Women’s age** |  |  |  |  |
| 15-19 |  | 1.00 |  | 1.00 |
| 20-24 |  | 1.27 [0.76, 2.10] |  | 1.27 [0.77, 2.11] |
| 25-29 |  | 2.66^***^ [1.63, 4.34] |  | 2.46^***^ [1.51, 4.02] |
| 30-34 |  | 3.20^***^ [1.88, 5.44] |  | 2.83^***^ [1.67, 4.80] |
| 35-39 |  | 4.21^***^ [2.31, 7.69] |  | 3.70^***^ [2.04, 6.72] |
| 40-44 |  | 4.24^***^ [2.35, 7.62] |  | 3.76^***^ [2.08, 6.77] |
| 45-49 |  | 4.55^***^ [2.17, 9.56] |  | 3.86^***^ [1.82, 8.17] |
| **Women’s educational level** |  |  |  |  |
| No education |  | 1.00 |  | 1.00 |
| Primary |  | 0.94 [0.76,1.16] |  | 1.12 [0.90, 1.40] |
| Secondary |  | 1.21 [0.96, 1.53] |  | 0.99 [0.78, 1.26] |
| Higher |  | 1.69^*^ [1.13, 2.53] |  | 1.30 [0.86, 1.98] |
| **Marital status** |  |  |  |  |
| Never in union |  | 1.00 |  | 1.00 |
| Married |  | 1.20 [0.81, 1.78] |  | 1.30 [0.86, 1.96] |
| Cohabiting |  | 0.95 [0.60, 1.50] |  | 1.14 [0.71, 1.83] |
| Widowed |  | 2.15^*^ [1.15, 4.03] |  | 2.64^**^ [1.39, 5.04] |
| Divorced |  | 0.88 [0.44, 1.79] |  | 1.03 [0.51, 2.11] |
| Separated |  | 0.73 [0.38, 1.39] |  | 1.00 [0.51, 1.95] |
| **Current working status** |  |  |  |  |
| No |  | 1.00 |  | 1.00 |
| Yes |  | 0.70^***^ [0.59, 0.83] |  | 0.73^***^ [0.62, 0.87] |
| **Parity** |  |  |  |  |
| Primiparity |  | 1.00 |  | 1.00 |
| Multiparity |  | 0.79 [0.48, 1.31] |  | 0.75 [0.46, 1.25] |
| Grandparity |  | 0.77 [0.43, 1.39] |  | 0.76 [0.43, 1.37] |
| **Height of the women (cm)** |  |  |  |  |
| Less than 145 |  | 1.00 |  | 1.00 |
| 145-149 |  | 0.52^*^ [0.28, 0.97] |  | 0.48^*^ [0.25, 0.91] |
| 150-154 |  | 0.66 [0.36, 1.22] |  | 0.56 [0.29, 1.07] |
| 155-159 |  | 0.69 [0.39, 1.23] |  | 0.55^*^ [0.30, 1.00] |
| 160+ |  | 0.69 [0.39, 1.22] |  | 0.51^*^ [0.28, 0.92] |
| **Currently breastfeeding** |  |  |  |  |
| No |  | 1.00 |  | 1.00 |
| Yes |  | 0.89 [0.71, 1.13] |  | 0.98 [0.78, 1.25] |
| **Sex of child** |  |  |  |  |
| Male |  | 1.00 |  | 1.00 |
| Female |  | 0.85 [0.72, 1.01] |  | 0.85 [0.72, 1.01] |
| **Age of child** |  |  |  |  |
| 0 |  | 1.00 |  | 1.00 |
| 1 |  | 0.84 [0.67, 1.04] |  | 0.86 [0.69, 1.06] |
| 2 |  | 0.42^***^ [0.31, 0.57] |  | 0.45^***^ [0.33, 0.61] |
| 3 |  | 0.39^***^ [0.29, 0.52] |  | 0.43^***^ [0.32, 0.57] |
| 4 |  | 0.52^***^ [0.39, 0.69] |  | 0.56^***^ [0.42, 0.74] |
| **Birthweight (kilograms)** |  |  |  |  |
| 2.5+ |  | 1.00 |  | 1.00 |
| Below 2.5 |  | 2.03^***^ [1.54, 2.69] |  | 1.96^***^ [1.48, 2.60] |
| **Diarrhoea in the last 2 weeks** |  |  |  |  |
| No |  | 1.00 |  | 1.00 |
| Yes |  | 1.10 [0.90, 1.35] |  | 1.14 [0.93, 1.39] |
| **Fever in the last 2 weeks** |  |  |  |  |
| No |  | 1.00 |  | 1.00 |
| Yes |  | 0.99 [0.79, 1.23] |  | 1.00 [0.80, 1.25] |
| **Cough in the last 2 weeks** |  |  |  |  |
| No |  | 1.00 |  | 1.00 |
| Yes |  | 0.86 [0.70, 1.07] |  | 0.96 [0.77, 1.19] |
| **Birth order** |  |  |  |  |
| First |  | 1.00 |  | 1.00 |
| Second |  | 1.08 [0.69, 1.69] |  | 1.15 [0.73, 1.81] |
| Third |  | 1.06 [0.65, 1.72] |  | 1.14 [0.70, 1.86] |
| Fourth or more |  | 1.05 [0.64, 1.74] |  | 1.15 [0.69, 1.93] |
| **Wealth index** |  |  |  |  |
| Poorest |  |  | 1.00 | 1.00 |
| Poorer |  |  | 1.37^*^ [1.05, 1.78] | 1.39^*^ [1.07, 1.81] |
| Middle |  |  | 1.61^***^ [1.25, 2.07] | 1.60^***^ [1.24, 2.07] |
| Richer |  |  | 1.93^***^ [1.43, 2.62] | 1.95^***^ [1.45, 2.64] |
| Richest |  |  | 2.23^***^ [1.64, 3.03] | 2.08^***^ [1.51, 2.87] |
| **Drinking water source** |  |  |  |  |
| Improved |  |  | 1.00 | 1.00 |
| Unimproved |  |  | 0.97 [0.78, 1.21] | 0.95 [0.77, 1.17] |
| **Toilet facility** |  |  |  |  |
| Improved |  |  | 1.00 | 1.00 |
| Unimproved |  |  | 1.00 [0.81, 1.23] | 1.01 [0.82, 1.24] |
| **Household size** |  |  |  |  |
| Small |  |  | 1.00 | 1.00 |
| Medium |  |  | 1.23^*^ [1.03, 1.47] | 1.05 [0.87, 1.27] |
| Large |  |  | 1.23 [0.96, 1.57] | 1.07 [0.83, 1.38] |
| **Place of residence** |  |  |  |  |
| Urban |  |  | 1.00 | 1.00 |
| Rural |  |  | 0.79^*^ [0.63, 0.99] | 0.80 [0.64, 1.01] |
| **Geographical subregions** |  |  |  |  |
| Southern |  |  | 1.00 | 1.00 |
| Central |  |  | 0.51^***^ [0.34, 0.76] | 0.55^**^ [0.36, 0.83] |
| Eastern |  |  | 0.30^***^ [0.21, 0.43] | 0.30^***^ [0.21, 0.43] |
| Western |  |  | 0.84 [0.61, 1.16] | 0.86 [0.62,1.19] |
| **Random effect model** |  |  |  |  |
| PSU variance (95% CI) | 1.524 [1.284, 1.808] | 1.474 [1.239, 1.754] | 1.483 [1.245, 1.767] | 1.485 [1.240, 1.780] |
| ICC | 0.316 | 0.309 | 0.311 | 0.311 |
| Wald chi-square | Reference | 279.46 (<0.001) | 264.46 (<0.001) | 562.47 (<0.001) |
| **Model fitness** |  |  |  |  |
| Log-likelihood | -14939.008 | -14446.562 | -14492.967 | -14098.28 |
| AIC | 29882.02 | 28965.12 | 29013.93 | 28292.56 |
| BIC | 29901.49 | 29315.7 | 29150.27 | 28760 |
| N | 125280 | 125280 | 125280 | 125280 |
| Number of clusters | 1608 | 1608 | 1608 | 1608 |

AOR= adjusted odds ratios; CI= Confidence Interval; ^*^ *p* < 0.05, ^**^ *p* < 0.01, ^***^ *p* < 0.001; 1.00 = Reference category; PSU=Primary Sampling Unit; ICC = Intra-Class Correlation; AIC = Akaike’s Information
